# Microbial diversity in the critically endangered Orinoco crocodile (*Crocodylus intermedius*): influence of body site and *Helicobacter* spp. on microbiota composition

**DOI:** 10.3389/fmicb.2025.1697111

**Published:** 2025-11-21

**Authors:** Loreley Castelli, María Alexandra García-Amado, Carla A. Rudolf, Monica Contreras, Ariel S. Espinosa-Blanco, Filipa Godoy-Vitorino

**Affiliations:** 1 Laboratorio de Microbiología y Salud de las Abejas, Departamento de Microbiología, Instituto de Investigaciones Biológicas Clemente Estable, Montevideo, Uruguay; 2 Centro de Investigaciones en Ciencias Ambientales, Instituto de Investigaciones Biológicas Clemente Estable, Montevideo, Uruguay; 3 Laboratorio de Fisiología Gastrointestinal, Centro de Biofísica y Bioquímica, Instituto Venezolano de Investigaciones Científicas, Caracas, Venezuela; 4 Laboratorio de Ecología y Genética de Poblaciones, Centro de Ecología, IVIC, Caracas, Venezuela; 5 Grupo de investigación Salus Conservatio, Programa de Biología, Universidad Internacional del Trópico Americano, UNITRÓPICO, Yopal, Colombia; 6 Departamento de Microbiología y Zoología Médica, Escuela de Medicina, Universidad de Puerto Rico, Recinto de Ciencias Médicas, San Juan, Puerto Rico

**Keywords:** *Crocodylus intermedius*, bacterial community, *Helicobacter* spp., *Campylobacter* spp., species extinction, captivity, body site crocodiles

## Abstract

**Introduction:**

The Orinoco crocodile (*Crocodylus intermedius*), a critically endangered species from Colombia and Venezuela Llanos, continues to face significant threats despite existing legal protections. Understanding the microbial diversity associated with this species, particularly in captive populations, can offer valuable insights into its health status and inform conservation strategies. In this study, we characterized the bacterial microbiota of *C. intermedius*, focusing on the influence of body site and the presence of *Helicobacter* spp. on microbial diversity.

**Methods:**

We collected oral and cloacal samples from five captive *C. intermedius* individuals and analyzed their bacterial microbiota using high-throughput sequencing techniques. The study specifically investigated how microbial diversity varies by body site and how the presence of *Helicobacter* spp. influences community structure and composition.

**Results:**

Oral samples exhibited higher microbial diversity compared to cloacal samples. This difference is likely attributable to greater environmental exposure and dietary variation affecting the oral cavity. The presence of Helicobacter spp. was associated with a marked reduction in bacterial richness and significant shifts in community composition. Samples positive for *Helicobacter* spp. were notably enriched in potentially pathogenic genera, including *Campylobacter* and *Escherichia*, suggesting a dysbiotic effect on the microbiota.

**Discussion:**

Our findings indicate that both body site and *Helicobacter* spp. presence play significant roles in shaping the microbial communities of *C. intermedius*. These results have important implications for reptile health management and zoonotic disease surveillance, as dysbiosis could compromise host health and facilitate pathogen transmission. Furthermore, this study underscores the role of reptiles as potential reservoirs for *Campylobacter* spp. and *Helicobacter* spp., highlighting the need for continued research into the microbial ecology of endangered species to guide conservation strategies and inform public health policies.

## Introduction

The Orinoco crocodile (*Crocodylus intermedius*) is one of the seven species of crocodiles in the world listed as critically endangered by the IUCN Red List of Threatened Species (Balaguera-Reina et al., 2018). Nowadays, the Orinoco crocodile is restricted to a few wild populations in the Venezuelan and Colombian Llanos (Seijas and Chávez, 2000; Balaguera-Reina et al., 2018). Although international trade of this species and its products have been banned by CITES (Appendix I: https://cites.org/eng) since mid-1970s (King et al., 1992), and it has also been given a protected status in both Venezuela and Colombia for more than 30 years along with the development of conservation programs, current surveys carried out in both countries continue to show a decreasing trend in their populations (Babarro, 2017; Espinosa-Blanco et al., 2017; Velasco et al., 2020).

The Orinoco crocodile conservation program in Venezuela involves both *in situ* and *ex situ* approaches. While *in situ* strategies consist of management of important natural populations of the species and their ongoing assessment and monitoring, *ex situ* efforts rely on captive breeding centers (CBCs), such as Masaguaral, Puerto Miranda, and UNELLEZ facilities, where individuals are produced and subsequently released to replenish wild populations (Hernández, 2007; Babarro, 2008). However, captivity poses some troubles, such as dietary changes, environmental stress, habitat homogenization, inbreeding and reduced genetic diversity (Jiang et al., 2025). In captivity, constant contact with human keepers provides increased opportunities for transmission of microbiota from host-associated sources, which are capable of colonizing the animals. Additionally, both a reduction in the diversity of diet items and antibiotic use in species living in captivity lead to a decrease in microbiota diversity compared to those living in the wild. Since these factors could affect the microbiota of animals, understanding the structure and assembly of the commensal microorganisms, as well as the effects of captivity, is crucial for improvements in animal health and reintroduction programs based on captive breeding (Williams et al., 2018; Tang et al., 2020).

Crocodilians are not the exception, as it has been previously suggested that captivity and handling could alter their bacterial community (Boede and Velasco, 1993; Rudolf et al., 2018). Nevertheless, studies on the gastrointestinal microbiota of crocodilians are scarce. Charruau et al. (2012) assessed the oral and cloacal microbiota of wild specimens of *Crocodylus acutus* and *C. moreletii* in Mexico through bacterial culture and chemical analyses, finding several potentially pathogenic bacterial species in both crocodilian species with no apparent signs of disease. A similar study was carried out on *C. palustris* in Iran, where DNA from the isolates was analyzed by 16S rRNA gene sequencing for bacterial identification (Gholamhosseini et al., 2021). More recently, Willson et al. (2019) and Siddiqui et al. (2023) characterized the gut microbiota of *C. porosus* using 16S rRNA-based metagenomic approaches and reported contrasting results in the composition and dominance of bacterial phyla throughout the gastrointestinal tract. To our knowledge, there’s only one study that assesses the bacterial community composition in health and disease states in crocodilians. It was conducted on *C. siamensis*, and the authors found clear changes in gut microbial composition between sick and healthy crocodiles (Lin et al., 2019). In *C. intermedius*, Rudolf et al. (2018) reported for the first time the presence of the genus *Helicobacter*, including *H. pylori* in cloacal and oral samples. In this study, we used the same samples from captive individuals used by Rudolf et al. (2018) to determine if the presence of *Helicobacter* spp. might affect the bacterial community.

## Materials and methods

### Sample collection

In July 2016, five adult Orinoco crocodiles (*C. intermedius*) from a Venezuelan CBC at University of Los Llanos (UNELLEZ; 08⁰36′45”N; 69⁰26′45.08”W) were captured and sexed following the methodology described in Espinosa-Blanco et al. (2017).

The oral and cloacal sterile cotton swab samples of the five individuals were collected at the CBC and frozen in liquid nitrogen until arrival at IVIC where they were stored at −80 °C until DNA extraction. All animals were captured and released under Venezuelan Ministry of Environment permits (0314 and DGDB00057).

### DNA extraction and sequencing

DNA extraction was performed as described in Rudolf et al. (2018). The 16S rRNA gene was amplified using universal V4 primers 515F (5’-GTGCCAGCMGCCGCGGTAA-3′) and 805R (5’-GGAC TACHVGGGTWTCTAAT-3′) following protocols from the Earth Microbiome Project.^1^ Sequencing was performed on an Illumina MiSeq platform. The demultiplexed amplicon data were deposited and quality-checked in QIITA (project ID 16059), and subsequently analyzed using QIIME2 version 2023.7 and RStudio.^2^

### Detection of *Helicobacter* spp.

The same DNA samples analyzed in our study had previously been tested for *Helicobacter* spp. by Rudolf et al. (2018), who performed PCR-based detection of genus-specific Helicobacter primers (16S rRNA gene, Germani et al., 1997) and *glmM* gene amplification for *H. pylori* (Kansau et al., 1996). This prior screening provided the *Helicobacter* spp. status (positive/negative) of each sample.

### Statistical analyses

#### Bioinformatic analysis

Illumina sequence reads were processed using R Studio Software version 4.3.2^3^ and Divisive Amplicon Denoising Algorithm 2 (DADA2) package version 1.12.1 (Callahan et al., 2016). Low quality raw reads were discarded from obtained data and primer sequences were removed using cutadapt (Martin, 2011). Then, reads were truncated to 140 bp. Quality filtering of reads with Phred scores <20. Amplicon sequence variants (ASVs) were inferred using DADA2 version 1.12.1 (Callahan et al., 2016), which corrects sequencing errors. Filtered based on length, representative sequences were obtained and denoised, and chimeric reads were removed. Then, paired reads were merged. Taxonomy was assigned to amplicon sequence variants (ASVs) with the SILVA_132 database via the assignTaxonomy function, and sequences identified as mitochondria, chloroplast, or eukaryotic were excluded using the subset_taxa function in the phyloseq package (version 1.28.0) (McMurdie and Holmes, 2013).

To facilitate the visualization of the barplots (relative ASVs abundance), we retained only ASVs that have at least 1% relative abundance in minimum 2 samples (“genefilter” package version 1.88.0 (Gentleman et al., 2024), “filterfun_sample” function). Alpha and beta diversity were calculated using the “Vegan” package (Oksanen et al., 2025) with the complete ASVs table. To evaluate alpha diversity, we calculated the number of observed ASVs, Shannon and Chao1 index (Anderson, 2006). Then, we evaluated beta diversity by using Bray–Curtis, UniFrac weighted (by the relative abundance of ASVs) and UniFrac Unweighted (presence/absence of ASVs) indexes (“vegdist” function) (Anderson, 2006). To test the effect of variables on community structure, we used permutational multivariate analysis of variance (ADONIS, “adonis” function) on beta diversity data. We then used the function “betadisper” to test for homogeneity of multivariate dispersions (Anderson, 2006; Anderson et al., 2006) and compared the distances of individual samples to group centroids in multidimensional space using “permutest.” The “metaMDS” function was used to plot ordinations. Finally, we evaluated the differences in the relative abundance of ASVs between groups using the DESeq software (Love et al., 2014). We used the Wald test in DESeq2 to determine if the estimated fold change of 16S rRNA genes was significantly different between sample groupings.

## Results

### *Crocodylus intermedius* microbiota

**Figure 1:**
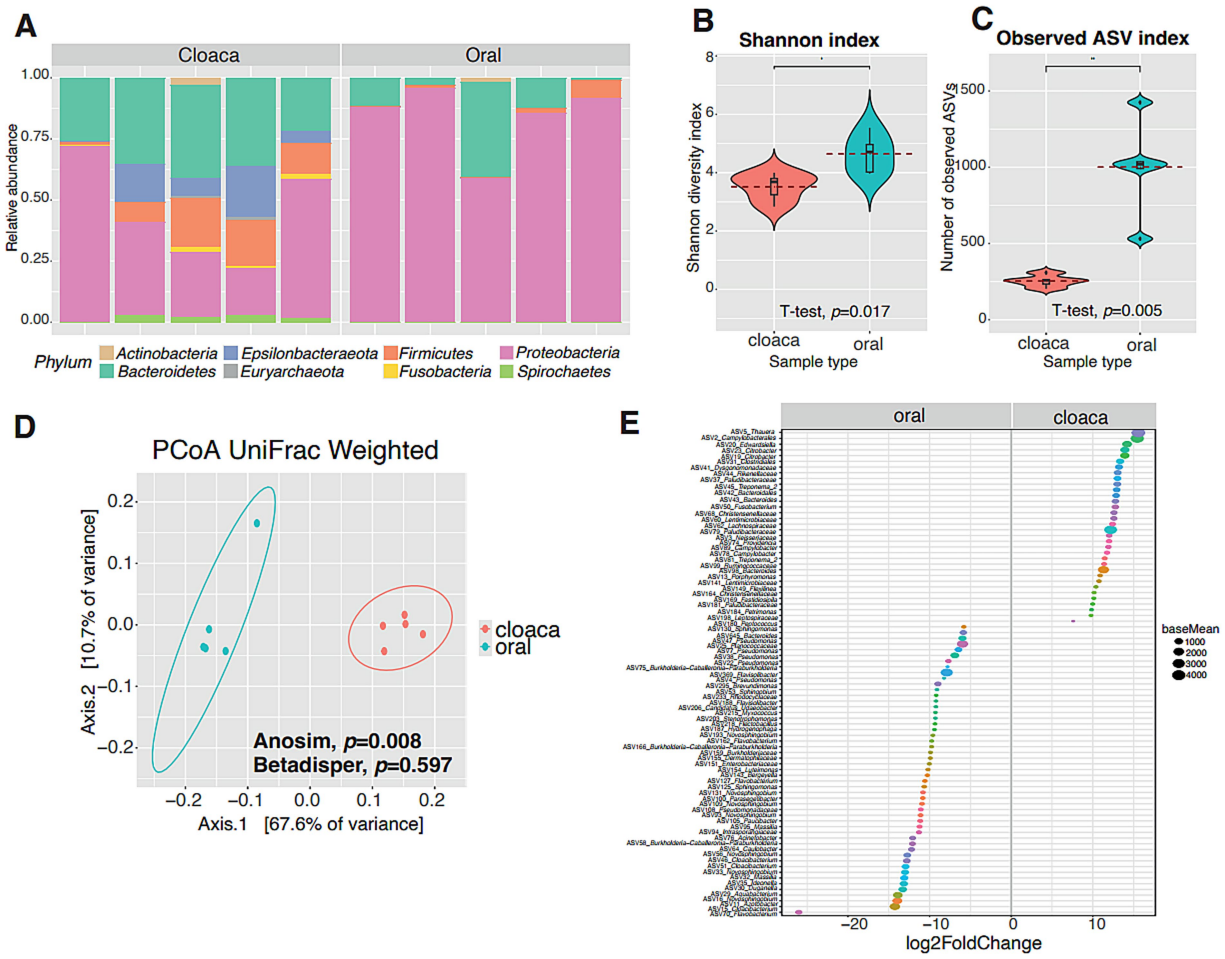
Bacterial microbiota of the Orinoco crocodile (*Crocodylus intermedius*) in cloacal and oral samples. **(A)** Relative abundance of bacterial phyla detected in *C. intermedius* cloacal and oral samples using high-throughput sequencing of the 16S rRNA gene (V4 region). Each bar represents an individual sample, and colors indicate different bacterial phyla. **(B)** Observed ASVs index reflecting species richness in each sample. **(C)** Shannon alpha-diversity index showing both richness and evenness of ASVs per sample. Results are shown as box shown as box plots, including median, 25 and 75% quartiles and outlier’s values. **(D)** Weighted UniFrac beta diversity, representing compositional differences between samples by PERMANOVA. **(E)** Comparison between crocodile cloacal and oral samples. Significant differential abundance results obtained with DESeq2 using the Wald test. A negative value of log2 fold changes means a decreased abundance in crocodile cloacal (increased in oral samples) and a positive value means an increased abundance in cloaca samples (decreased in oral samples). Asterisks indicate statistically significant differences in pairwise comparisons (*p* < 0.05). Five *C. intermedius* per sample type were individually analyzed.

After quality filtering, a total of 1,631,768 reads were retained across 10 samples (5 oral and 5 cloacal), yielding an average of 163,177 reads per sample (range: 121,389–206,077) and identifying 1,300 unique ASVs. The most abundant phyla among the samples studied were *Proteobacteria*, *Bacteroidetes*, *Epsilonbacteraeota*, *Firmicutes*, *Spirochaetes*, *Euryarchaeota*, *Fusobacteria,* and *Actinobacteria* (Figure 1A).

The oral samples showed significantly greater richness and diversity compared to the cloacal samples, according to the observed ASVs and Shannon index (T-test *p* = 0.0059 and *p* = 0.017, respectively; Figures 1B,C).

Regarding beta diversity indexes, the microbiota diversity of the oral and cloacal samples was significantly different based on the UniFrac Weighted index (PERMANOVA *F* = 14.8, *p* = 0.008). Although the other indexes, Bray-Curtis and UniFrac Unweighted, were also significant, they were not considered because the dispersion between individuals was not similar (Beta dispersion *p* = 0.026 and *p* = 0.027, respectively; Figure 1D).

DESeq2 results at the genus level show clear differences between oral and cloacal samples. These results indicate that 84 ASVs showed differential abundance between oral and cloacal samples: 34 were more abundant in cloacal samples, while 50 were more abundant in oral samples. Among the 50 ASVs that were more abundant in oral samples compared to cloacal samples, taxa from the genus *Pseudomonas* and the family *Enterobacteriaceae* were especially enriched (Figure 1E).

### Impact of *Helicobacter* spp. presence on the microbiota of oral and cloacal samples of *Crocodylus intermedius*

**Figure 2:**
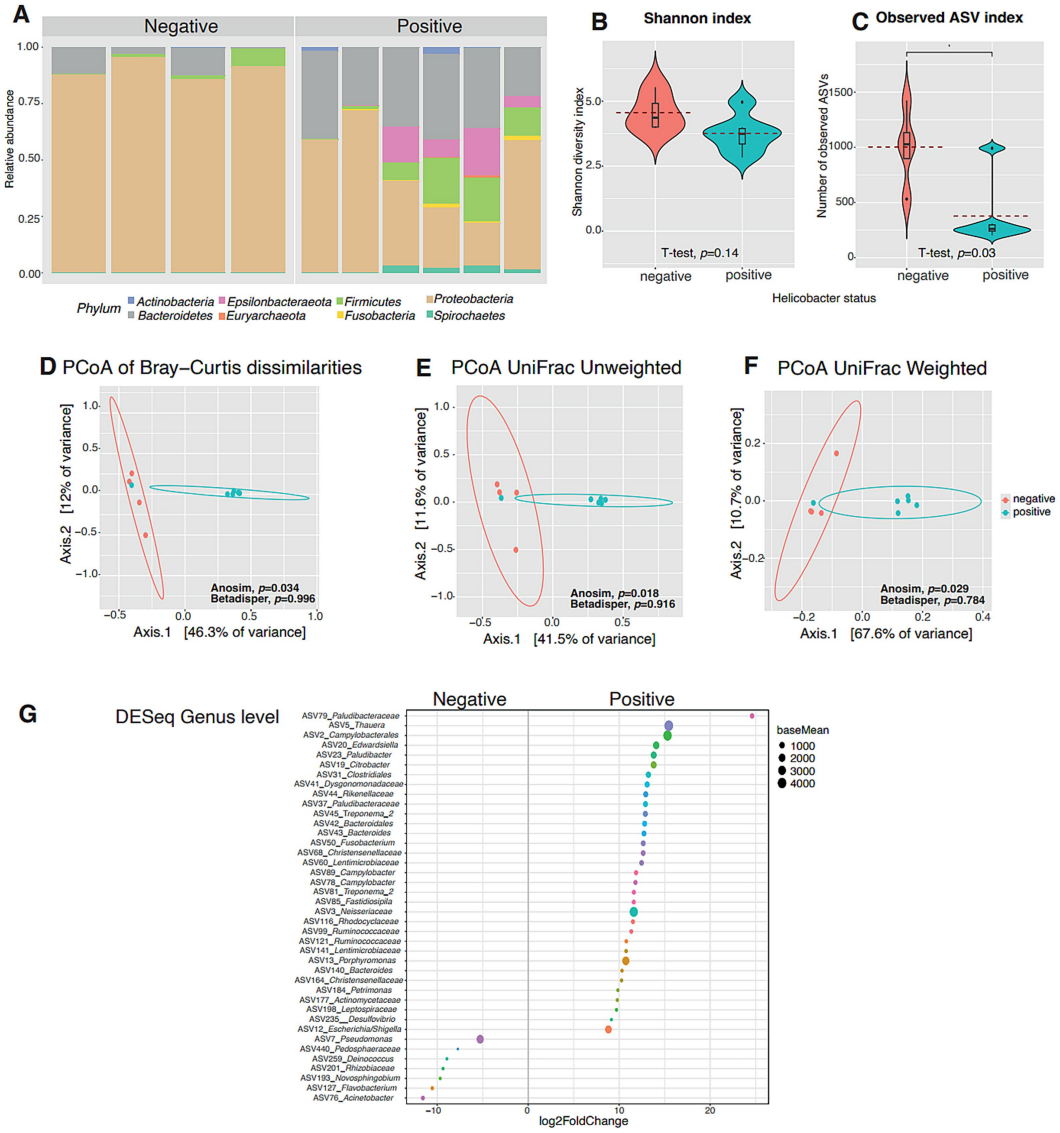
Bacterial microbiota of *Crocodylus intermedius* samples grouped by *Helicobacter* spp. detection status (negative vs. positive). **(A)** Relative abundance of bacterial phyla in *C. intermedius* samples, testing negative or positive for *Helicobacter* spp., based on high-throughput sequencing of the 16S rRNA gene (V4 region). Each bar represents an individual sample, and colors indicate distinct bacterial phyla. **(B)** Observed ASVs index, reflecting the richness of amplicon sequence variants in each sample. **(C)** Shannon alpha-diversity index, representing both richness and evenness of ASVs. Results are shown as box shown as box plots, including median, 25 and 75% quartiles and outlier’s values. **(D)** Bray–Curtis dissimilarity index, showing compositional dissimilarities between groups by PERMANOVA. **(E,F)** Unweighted and Weighted UniFrac beta diversity indices, illustrating phylogenetic differences in community composition. **(G)** Results of differential abundance analysis obtained with DESeq2 using the Wald test. A negative value of log2 fold changes means a decreased abundance in crocodile *Helicobacter* spp. positive (increased in negative samples) and a positive value means an increased abundance in positive samples (decreased in negative samples). Asterisks indicate statistically significant differences between pairwise comparisons (*p* < 0.05).

The presence of *Helicobacter* spp. was associated with a significant decrease in microbial richness as indicated by the observed ASVs index (*t*-test, *p* = 0.032). However, no significant differences in diversity were observed between the groups based on the Shannon index (*t*-test, *p* = 0.14; Figures 2B–C). Overall, *Helicobacter* spp. positive samples exhibited lower bacterial diversity and richness compared to *Helicobacter* spp. negative samples.

Regarding beta diversity indexes, the microbiota composition of *Helicobacter* spp. negative and *Helicobacter* spp. positive samples differed significantly according to the UniFrac Weighted, UniFrac Unweighted, and Bray-Curtis indexes (PERMANOVA, *p* < 0.05 in all cases; Figures 2D–F). The dispersion between individuals was similar across groups (Beta dispersion, *p* > 0.05 in all cases).

Clustering analysis showed that samples clearly grouped according to sampling site (cloacal *vs*. oral) and *Helicobacter* spp. status (positive *vs*. negative; data not shown).

At the genus level, the presence of *Helicobacter* spp. had a clear effect on the microbiota, as revealed by DESeq2 analysis (Figure 2G). These results indicate that 40 ASVs showed differential abundance between *Helicobacter* spp. positive and *Helicobacter* spp. negative samples: 4 were more abundant in *Helicobacter* spp. negative samples, while 37 were more abundant in *Helicobacter* spp. positive samples. Among the 37 ASVs that were more abundant in *Helicobacter* spp. positive samples compared to *Helicobacter* spp. negative samples, taxa such as *Campylobacter* and *Escherichia* spp. were especially enriched (Figure 1E).

## Discussion

In this study, we characterized the bacterial microbiota of *Crocodylus intermedius* from Venezuela, highlighting the influence of body site and the presence of *Helicobacter* spp. on microbial diversity and structure. Our results show that oral samples consistently exhibited higher microbial diversity compared to cloacal samples, likely due to the oral’s broader exposure to aquatic environmental microbes (Keenan et al., 2013). This finding reflects the idea that different body areas provide unique niches for microbial growth.

In *C. intermedius*, the dominant bacterial phyla are Proteobacteria, Bacteroidetes, Firmicutes, and Epsilonbacteraeota, but the proportion of each phylum is dynamic (Figures 1, 2). In oral samples, the Proteobacteria is the predominant phylum (Figure 1A). Similar results had been reported in oral samples of other Crocodilia species in captivity, like *Alligator mississippiensis* (Keenan et al., 2013) and *C. porosus* (Siddiqui et al., 2023), and in cloacal samples of diseased *C. siamensis* (Lin et al., 2019). In our study, the Proteobacteria abundance is associated with other pathogen genera like *Pseudomonas*, *Acinetobacter,* and *Burkholderia*, as well as environmental bacteria like *Azotobacter*, *Aquabacterium,* and *Duganella* (Figure 1E). *Pseudomonas genus* was reported in oral samples of captive *Crocodylus porosus* (Siddiqui et al., 2023). The genus *Acinetobacter* was reported in cloacal samples of captive newborn Chinese Alligators (Zhang et al., 2023), in fecal samples of Crocodile Lizards (*Shinisaurus crocodilurus*) (Tang et al., 2020), and in fecal samples of captive *C. intermedius* in Colombia (Polanco Rodríguez, 2023). Shin et al. (2015) report that the abundance of Proteobacteria in the gut may reflect dysbiosis or an unstable gut microbial community structure. Although the sampled animals in this study did not show signs of disease, the presence of pathogenic genera may indicate an alteration of their microbiota.

In cloacal samples, the predominant phyla are Proteobacteria and Bacteroidetes (Figure 1A). Similar results had been reported in cloacal samples of other reptile species in captivity, like *Shinisaurus crocodilurus* (Jiang et al., 2017). However, one study made in Colombia with captive *C. intermedius* reports Firmicutes, Bacteroidetes, and Proteobacteria as predominant phyla (Polanco Rodríguez, 2023). In this study, the predominant genera in cloacal samples are *Edwardsiella*, *Campylobacter* and, *Providencia* from Proteobacteria, and *Bacteroides* from Bacteroidetes (Figure 1E). *Edwardsiella* and *Bacteroides* have been reported in cloacal samples of diseased *Crocodylus siamensis* (Lin et al., 2019), and *Edwardsiella* was reported in fecal samples of captive *C. intermedius* in Colombia (Polanco Rodríguez, 2023).

The presence of *Helicobacter* spp. significantly decreased microbiota richness in the crocodiles, suggesting a potential dysbiotic effect, in line with previous studies suggesting that *Helicobacter* spp. can cause dysbiosis (Jiang et al., 2017). Although no significant differences in diversity were observed between *Helicobacter* spp. positive and negative samples, the reduced richness in colonized individuals could still suggest underlying disruptions to gut health.

The impact of *Helicobacter* spp. on the microbiota parallels findings in other reptiles (Gilbert et al., 2014, 2017). While no studies to date have demonstrated a causal link between reptile associated *Helicobacter* spp. and gastrointestinal disease or microbiota imbalance, it could be hypothesized that under certain conditions, these bacteria may contribute to dysbiosis, potentially influencing their relative abundance. Future research is required to evaluate this possibility.

Additionally, our study aligns with previous research showing reptiles as reservoirs for *Campylobacter* spp. and *Helicobacter* spp. *Campylobacter fetus* and related species, such as *C. iguaniorum* and *C. geochelonis*, have been isolated from various reptiles and associated with both reptilian and human infections (Harvey and Greenwood, 1985; Tu et al., 2004; Benejat et al., 2014). These species, particularly *Helicobacter* spp., exhibit high host specificity, with distinct lineages identified in lizards and chelonians, suggesting long-term coevolution with their reptilian hosts (Gilbert et al., 2014, 2017).

Beyond their role in gastrointestinal health, *Campylobacter* species may also influence host neurobiology in mammals. *Campylobacter jejuni*, a well-documented enteropathogen, is highly sensitive to environmental stressors such as nutrient limitation, heat, dehydration, low pH, and UVB exposure (Goehler et al., 2008). Despite these challenges, it remains a significant foodborne pathogen capable of impacting host behavior. Studies in mice have shown that *C. jejuni* infection induces anxiety-like behavior even in the absence of overt gastrointestinal illness (Lyte et al., 1998). This suggests a potential gut-brain axis interaction, where microbial infections could alter host neurological responses. Although not the primary focus of this study, the potential neurobehavioral effects of microbial infections in reptiles remain largely unexplored. Investigating how the reptilian microbiota may influence host physiology beyond the gut could provide valuable insights into host–microbe interactions and their broader implications for reptile health and behavior.

The presence of these pathogens in reptiles highlights their potential as reservoirs for zoonotic diseases, particularly among immunocompromised individuals. Evidence from multiple studies indicates that *Campylobacter* spp. can colonize the reptilian gut and be transmitted to humans through fecal contamination or direct contact. It is plausible that *Helicobacter* spp. may follow similar transmission pathways; however, direct evidence of its occurrence in reptiles is currently scarce (Masila et al., 2020; McWhorter and Whiley, 2025). Moreover, the adaptation of these species to specific ecological niches within their hosts may provide them with a competitive advantage, influencing microbial community dynamics and host health.

Although few studies have characterized the microbiota of *Crocodylus intermedius*, our findings partially align with patterns observed in other reptiles, particularly the dominance of Proteobacteria, Bacteroidetes, and Firmicutes in oral and cloacal samples (Keenan et al., 2013; Siddiqui et al., 2023; Lin et al., 2019; Jiang et al., 2017). Notably, the associations between *Helicobacter* spp. colonization and reduced microbial richness, along with the specific composition of pathogen-associated genera such as *Pseudomonas*, *Acinetobacter*, *Edwardsiella*, and *Campylobacter*, appear unique to *C. intermedius*, highlighting the novelty and significance of our study in advancing the understanding of reptile gut microbiota and potential pathogen reservoirs.

In conclusion, this study underscores the importance of body site and *Helicobacter* spp. presence in shaping reptilian microbiota. It also emphasizes the role of reptiles as potential reservoirs for *Campylobacter* spp. and *Helicobacter* spp., with implications for both reptile health and zoonotic disease transmission.

Future research should focus on long-term monitoring of microbiome dynamics in *Crocodylus intermedius*, investigating the functional roles of these microbes, and elucidating the specific relationships between microbiome composition, *Helicobacter* spp. colonization, and crocodile health. Understanding these aspects will inform reptile conservation, enhance veterinary care, and strengthen public health strategies.

## Data Availability

The raw sequencing data is deposited in the NCBI Sequence Read Archive (SRA) under the BioProject accession number PRJNA1313126. The data are publicly available at: https://www.ncbi.nlm.nih.gov/bioproject/PRJNA1313126.

## Footnotes

1
https://earthmicrobiome.org/protocols-and-standards/

2
https://www.r-project.org/

3
http://www.rstudio.com
