# Correction: Microbial diversity in the critically endangered Orinoco crocodile (*Crocodylus intermedius*): influence of body site and *Helicobacter* spp. on microbiota composition

**DOI:** 10.3389/fmicb.2026.1816066

**Published:** 2026-03-12

**Authors:** Loreley Castelli, María Alexandra García-Amado, Carla A. Rudolf, Monica Contreras, Ariel S. Espinosa-Blanco, Filipa Godoy-Vitorino

**Affiliations:** 1 Laboratorio de Microbiología y Salud de las Abejas, Departamento de Microbiología, Instituto de Investigaciones Biológicas Clemente Estable, Montevideo, Uruguay; 2 Centro de Investigaciones en Ciencias Ambientales, Instituto de Investigaciones Biológicas Clemente Estable, Montevideo, Uruguay; 3 Laboratorio de Fisiología Gastrointestinal, Centro de Biofísica y Bioquímica, Instituto Venezolano de Investigaciones Científicas, Caracas, Venezuela; 4 Laboratorio de Ecología y Genética de Poblaciones, Centro de Ecología, IVIC, Caracas, Venezuela; 5 Grupo de investigación Salus Conservatio, Programa de Biología, Universidad Internacional del Trópico Americano, UNITRÓPICO, Yopal, Colombia; 6 Departamento de Microbiología y Zoología Médica, Escuela de Medicina, Universidad de Puerto Rico, Recinto de Ciencias Médicas, San Juan, Puerto Rico

**Keywords:** *Crocodylus intermedius*, bacterial community, *Helicobacter* spp., *Campylobacter* spp., species extinction, captivity, body site crocodiles

An incorrect **Funding** statement was provided. The funder National Institutes of Health, NIGMS # PR-INBRE P20GM103475–22 and PR-CMS 1P20GM156713–01, to Filipa Godoy Vitorino was erroneously included.

The correct statement is

**Funding**

The author(s) declared that financial support was received for this work and/or its publication. This work was funded by an Instituto Venezolano de Investigaciones Científicas grant to MG-A. and MC. Idea Wild was funded a computer to MG-A. The field work was supported by IUCN-SSC Crocodile Specialist Group Student Research Assistance Scheme, the Scott Neotropical Fund, Cleveland Metroparks, and the Mohamed bin Zayed Species Conservation Fund to AE-B. Partial support including time for revision of analyses and publication (FGV) was given by RCMI Center for Collaborative Research in Minority Health and Health Disparities (2U54MD007600).

The original version of this article has been updated.

